# Demographic predictors on traditional prenatal service uptake among rural Zimbabwean pregnant women

**DOI:** 10.4314/ahs.v25i3.9

**Published:** 2025-09

**Authors:** Taruvinga Muzingili, Nicole Chatindo, Lizzy Zinyemba

**Affiliations:** 1 Department of Social Work, Midlands State University; 2 Department of Social Work, Women's University in Africa; 3 Department of Social Work, Bindura University of Science Education

**Keywords:** Traditional, prenatal care, Demographic, Rural Zimbabwe

## Abstract

**Background:**

This study aimed to identify demographic predictors influencing the uptake of traditional prenatal care among pregnant women in rural Zimbabwe to inform maternal health policies and programming.

**Methods:**

A cross-sectional study was conducted in Goromonzi District, focusing on Wards 1, 3, and 6. A census approach recruited 867 pregnant women identified through community health workers. Data were collected using questionnaires and analyzed using binary logistic regression to evaluate the influence of demographic predictors, including age, education, income, employment, parity, marital status, religion, and health complications.

**Results:**

Half (50%) of respondents reported using traditional prenatal services, with 80% preferring a combination of traditional and conventional care. Significant predictors included African Traditional Religion (ATR) (OR = 19.144, p = 0.008), parity (OR = 12.962, p = 0.004), low income (OR = 9.991, p = 0.004), informal employment (OR = 5.134, p < 0.001), and primary education (OR = 5.966, p = 0.006). The model explained 48.4% of the variance in traditional care adoption (Nagelkerke R^2^ =0.484).

**Conclusion:**

Integrated maternal health approaches respecting cultural practices, subsidized maternal services, and collaboration with traditional birth attendants could enhance maternal health outcomes in rural Zimbabwe.

## Introduction

Prenatal care is vital for safeguarding maternal and child health, yet disparities in access remain a persistent issue, particularly in low and middle-income countries (LMICs). Each year, over 300,000 women die from preventable pregnancy-related causes, with 94% of these deaths occurring in LMICs^1^. In rural Zimbabwe, limited healthcare infrastructure, socio-economic barriers, and cultural beliefs significantly hinder access to conventional prenatal care^1^,^2^. Consequently, many women rely on traditional prenatal services provided by traditional birth attendants (TBAs), who offer culturally sensitive and accessible care^3^.

However, the reliance on traditional care raises questions about its effectiveness and the demographic factors influencing its adoption. This study aims to fill the knowledge gap by identifying demographic predictors of traditional prenatal care use, providing insights to inform maternal health policies and programming in rural Zimbabwe. Understanding these predictors provides valuable insights for policy makers and healthcare providers. By identifying key demographic predictors of traditional prenatal care utilization, the findings can inform targeted prenatal care policies and programming, promoting the integration of culturally relevant traditional practices within existing healthcare frameworks to improve maternal health outcomes.

Traditional prenatal services and methods remain critical in rural areas, mainly where access to modern healthcare is limited. In Nigeria, traditional birth attendants (TBAs) provide massages, herbal remedies, and spiritual guidance to pregnant women, especially in rural communities where the healthcare infrastructure is weak^5^. Similarly, in Tanzania^6^, Zimbabwe^4^, and Peru^7^, TBAs offer herbal treatments and advice rooted in indigenous knowledge systems, with many women relying on them due to affordability and cultural familiarity^2^. In India, rural women often use herbal tonics, dietary restrictions, and home remedies during pregnancy, guided by traditional healers or older women in the community. These practices are deeply tied to cultural beliefs and are particularly prevalent in tribal areas^8^. In South Africa, traditional prenatal care includes the use of medicinal plants to prevent complications and rituals to protect both mother and baby from perceived spiritual harm. This is common among Zulu and Xhosa communities in rural settings^9^. In Mexico's rural regions, midwives provide physical care and spiritual and emotional support during pregnancy, often incorporating rituals and herbal remedies to ensure a safe delivery^10^,^11^. These examples highlight the widespread reliance on traditional prenatal services in rural areas globally, particularly where cultural practices and healthcare inequties intersect.

There is minimal literature specifically addressing how demographic characteristics influence the adoption of traditional prenatal healthcare services, as most studies focus on the utilization of conventional prenatal care services. However, insights can be drawn from existing research on conventional care, particularly in rural settings across both developed and developing countries, to understand broader patterns in healthcare utilization. A study in Nigeria on age shows that younger women, especially adolescents, are less likely to utilize formal prenatal care due to limited knowledge and social barriers^12^. This trend may extend to traditional care, where older women with more significant community ties and cultural experience may prefer traditional services. In rural India, education significantly predicts prenatal care utilization, with women who have secondary or higher education being more likely to seek conventional prenatal care compared to those with little or no education^13^. This suggests that lower education levels could drive reliance on traditional services due to limited awareness of modern alternatives. In Kenya, Marital status is another key determinant, as married women tend to access prenatal care more frequently than unmarried women, often due to spousal or family support^14^.

In rural Bangladesh, income and employment status are strong predictors, with wealthier women and those in formal employment more likely to use modern maternity services^15^. Conversely, women in informal or subsistence employment, common in rural areas, may rely on traditional care due to financial constraints. Religion and ethnicity also influence healthcare choices. In rural Uganda, women from minority ethnic groups and those practicing traditional religions are more inclined to use traditional prenatal care, partly due to cultural norms and mistrust of formal systems^16^. Similarly, in the United States, studies among African American and Native American populations in rural areas highlight the role of cultural beliefs and racial discrimination in shaping preferences for traditional or community-based care^17^. While most studies focus on conventional services, these patterns suggest that demographic factors such as age, education, marital status, income, religion, and ethnicity strongly influence prenatal care choices, including the adoption of traditional services, particularly in rural and underserved areas.

## Material and Methods

### Design and setting

A cross-sectional study was conducted in Goromonzi rural district, Zimbabwe, a region predominantly inhabited by Shona-speaking people. The district is characterized by limited health infrastructure, with pregnant women often traveling long distances to access health services. The study focused on Wards 1, 3, and 6, representing typical rural settings with high reliance on traditional prenatal services due to accessibility challenges. The study aimed to identify demographic predictors influencing the uptake of traditional prenatal services among pregnant women in this region.

### Respondents

A census approach was adopted, targeting all pregnant women in Wards 1, 3, and 6, identified through community health workers. Inclusion criteria required women to be registered with community health workers as pregnant, regardless of age. Since Zimbabwean law mandates pregnant women to register with formal healthcare centers, the respondents included those who may or may not use conventional prenatal care services. This ensured a comprehensive sample of women with varying service preferences. The final sample consisted of women identified during community health outreach programs who consented to participate in the study.

### Measurements

The study examined demographic characteristics as independent variables. Continuous variables included age (in years), educational level (years of schooling), parity (number of previous pregnancies), and income level. Categorical variables included religion (African Traditional Religion, Christianity, Islam, others), employment status (binary: employed/unemployed), marital status (binary: married/unmarried), and health status (presence of pregnancy-related complications: yes/no/not sure). The dependent variable used traditional prenatal services, measured in binary (yes or no). These variables were informed by existing literature on predictors of healthcare utilization.

### Data collection

Data were collected using a researcher-designed questionnaire covering demographics and traditional prenatal service use. All questions were closed-ended to ensure consistency. Six trained research assistants administered the questionnaires in community settings and conventional health facilities. Self-administered questionnaires were made available in the local language for respondents who were illiterate in Shona. Research assistants assisted respondents who required help due to literacy challenges. Data collection spanned January 2023 to November 2024, ensuring adequate time for recruitment and capturing seasonal variations in prenatal care practices. The questionnaire was pretested with 20 women from a non-study ward to ensure clarity, reliability, and cultural relevance.

### Statistical analysis

Binary logistic regression was used to assess the relationship between demographic factors and the utilization of traditional prenatal services, with the dependent variable coded as binary (yes/no). Descriptive statistics, including frequencies and proportions, were used to summarize the demographic characteristics of respondents and their utilization of prenatal care services. Odds ratios (OR) and 95% confidence intervals (CIs) were calculated to determine the strength and significance of predictors, with a p-value of <0.05 considered statistically significant. Analysis was conducted using SPSS version 27.

## Results

### Demographic information

The respondents ranged from 16 to 46 years, with a mean age of 31.03 (SD = 8.678). The skewness value (-0.152) indicates a nearly symmetrical age distribution. Gestation ranged from 1 to 8 months, with a mean gestational age of 4.49 months (SD = 2.338), suggesting that most respondents were in mid-pregnancy. The distribution is nearly symmetrical (skewness = 0.025). Years of education ranged from 1 to 13, with a mean of 6.78 years (SD = 3.129). The positive skewness (0.250) indicates a slight concentration of respondents with lower education levels. Income ranged from $0 to $150, with a mean of $39.57 (SD = 33.640). The high skewness (1.970) suggests that most respondents earned lower incomes, with a few outliers earning substantially more. The number of previous pregnancies ranged from 0 to 7, with a mean parity of 2.00 (SD = 1.881). The positive skewness (0.820) shows that most women had fewer pregnancies, with fewer cases of higher parity. Of 867 respondents, 74% of respondents were married (n = 639), while 26.3% were unmarried (n = 228). 26.1% were employed (n = 226), while the majority (74%) were unemployed (n = 641). Christianity was the most common religion (56%, n = 489), followed by African Traditional Religion (ATR) at 31% (n = 269). Islam accounted for 9% (n = 75), and other religions comprised 4% (n = 34). 9% of the women (n = 78) reported experiencing pregnancy-related complications, 37% answered no (n=37), while the majority 54% (n = 468) reported no complications. These frequencies highlight the predominance of married, unemployed women, with most identifying as Christians and a significant proportion following ATR. Additionally, more than half of the respondents reported no health complications during pregnancy.

### Use of traditional prenatal services

**Figure 1:**
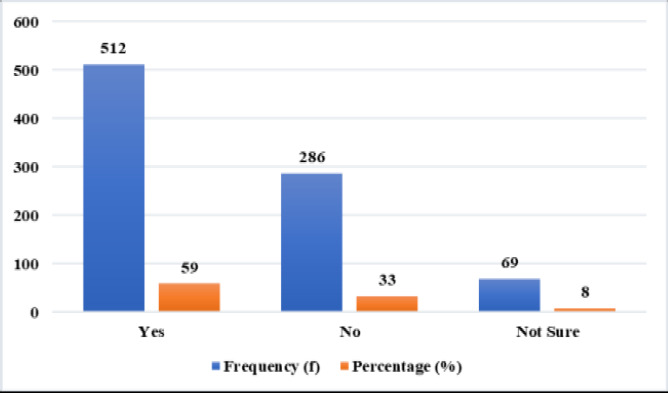
Utilisation of traditional prenatal services by pregnant women

Before assessing the influence of demographic factors on the utilization of traditional prenatal care services, respondents were first asked if they have used those services. Figure 1 below shows the response from the respondents

In Figure 1, the surveyed pregnant women were asked whether they used traditional prenatal services, with responses categorized as “Yes,” “No,” and “Not sure.” Of the total respondents, 50% (n=512) answered “Yes,” indicating more than half of the women reported using traditional prenatal services during their pregnancies. Of the total (n=867), 33% (n=286) responded “No,” demonstrating that a significant proportion of women did not utilize these services. Finally, 8% (n=69) answered “Not sure,” which may reflect a misunderstanding or lack of clarity regarding what constitutes traditional prenatal services. Overall, Figure 1 shows that a notable number of pregnant women use traditional prenatal services, but there is some ambiguity among respondents regarding what these services entail.

**Figure 2:**
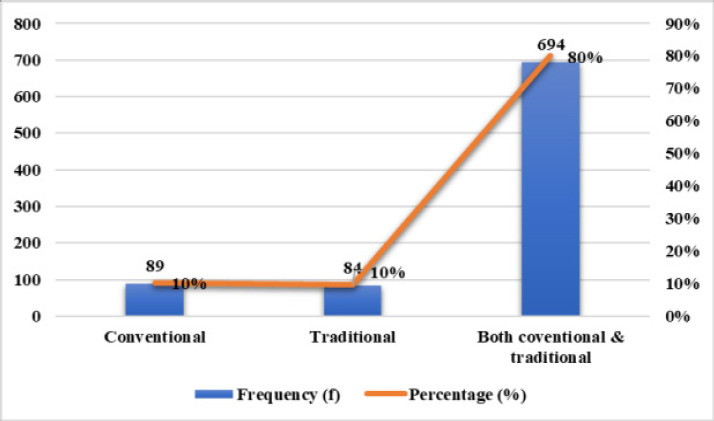
Pregnant women's preferred prenatal services

In Figure 2 below, the pregnant women's preferred prenatal services are assessed as indicated below:

Figure 2 examines the types of prenatal services preferred by pregnant women, with options categorized as “Conventional,” “Traditional,” and “Both.” The results show that 10% (n=89) preferred conventional services exclusively, indicating reliance on modern healthcare systems. Similarly, 10% (n=84) preferred traditional services exclusively, demonstrating that a smaller proportion rely solely on traditional methods. However, the largest group, 80% (n=694), preferred a combination of conventional services, indicating a blended approach to prenatal care. Many pregnant women prefer combining conventional prenatal services, reflecting a trend of integrating both systems for prenatal care.

### Demographic factors as predictors to utilisation of traditional prenatal services

The influence of pregnant women's demographic characteristics is assessed using binary logistic regression. Table 1 below shows the binary model summary and model fit test:

**Table 1:** Binary Logistic Regression model summary and fit test

Model Summary			Hosmer and Lemeshow Test			
Step	-2 Log likelihood	Cox & Snell R Square	Nagelkerke R Square	Chi-square	df	Sig.
1	114.317^a^	.335	.484	309.304	8	.897

The Hosmer and Lemeshow Test was used to assess the model's goodness of fit. The test produced a chi-square value of 309.304 with a significant value of 0.897. Since the p-value is greater than the significance threshold 0.05, the null hypothesis—that the model fits the data well—could not be rejected. This indicates that the model is a good fit for the data and sufficiently predicts the adoption of traditional prenatal services. The model summary explains how well the demographic predictors explain the variation in adopting traditional prenatal services. The Nagelkerke R^2^ value was 0.484, indicating the model explained approximately 48.4% of the variation in traditional care adoption. This suggests a moderate explanatory power of the model. The Cox & Snell R^2^ value was slightly lower at 0.335, reflecting the limitation of this measure, as it cannot reach a maximum value of 1. Additionally, the -2 Log Likelihood value of 114.317 suggests that the model has a reasonable fit, with lower values indicating better performance. Following the model summary, Table 2 below shows the influence of individual demographic variables used in binary logistic regression:

**Table 2:** Influence of individual demographic variable on utilisation of traditional prenatal services among pregnant women

		B	S.E.	Wald	df	Sig.	Exp(B)	
Step 1^a^	Age	.102	.018	31.090	1	.000	1.108	
	Gestation length	-.055	.035	2.440	1	.118	.946	
	Marital_(Single)	.286	.260	1.203	1	.033	1.752	
	Education_primary education	.834	.036	9.926	1	.006	5.966	
	Income_low income	7.009	.003	8.521	1	.004	9.991	
	Employment (informal)	2.008	.264	57.947	1	.000	5.134	
	Parity	1.638	.074	21.270	1	.004	12.962	
	Religion			.091	3	.993		
	ATR	20.392	.683	23.762	1	.008	19.144	
	Christianity	2.308	.013	.098	1	.998	.017	
	Islam	-.784	.516	.453	1	.561	.057	
	Health status (complications)	1.231	.093	5.612	1	.003	4.561	
	Constant	-22.807	.683	.000	1	.997	.000	

The analysis of individual predictors revealed several significant relationships. Age was a significant predictor, with a B coefficient of 0.102 (p < 0.001) and an odds ratio (Exp(B)) of 1.108, indicating that each additional year of age increased the odds of adopting traditional prenatal services by 10.8%. Marital status also emerged as a significant predictor, with single women being 1.752 times more likely to adopt traditional prenatal services compared to married women (B = 0.286, p = 0.033). Similarly, education level was influential: Women with only primary education were 5.966 times more likely to adopt traditional prenatal services than those with secondary or higher education (B = 0.834, p = 0.006). Income, specifically low income, was another strong predictor. Women in the low-income category were 9.991 times more likely to adopt traditional prenatal services compared to those in higher income brackets (B = 7.009, p = 0.004). Employment type also played a significant role, with women in informal employment being 5.134 times more likely to adopt traditional prenatal services than those in formal employment (B = 2.008, p < 0.001). Parity, which measures the number of previous pregnancies, was also a significant predictor, with women of higher parity being 12.962 times more likely to adopt traditional prenatal services (B = 1.638, p = 0.004).

Religion was also a significant factor. Women practicing African Traditional Religion (ATR) were 19.144 times more likely to adopt traditional prenatal services compared to women identifying with other religions (B = 20.392, p = 0.008). However, other religious categories, including Christianity and Islam, were not significant predictors of traditional prenatal service adoption. Lastly, health complications during pregnancy were found to be a significant predictor, with women experiencing complications being 4.561 times more likely to adopt traditional prenatal services (B = 1.231, p = 0.003). Some predictors, however, were not statistically significant. For example, gestation length (B = -0.055, p = 0.118) did not significantly predict traditional prenatal service adoption. Similarly, the religious categories of Christianity (p = 0.998) and Islam (p = 0.561) were not significant predictors within the model.

In summary, the analysis identified age, marital status, education, income, employment, parity, religion (ATR), and health complications as significant predictors of traditional prenatal service adoption. The strongest predictors, based on odds ratios, were African Traditional Religion (Exp(B) = 19.144), parity (Exp(B) = 12.962), and low income (Exp(B) = 9.991). These findings suggest that demographic factors such as religion, socioeconomic status, and maternal history play a critical role in influencing the adoption of traditional prenatal services.

## Discussion

The findings from this study highlight the significant role demographic factors play in shaping the adoption of traditional prenatal services among pregnant women in rural Zimbabwe. Approximately 50% of participants reported using traditional prenatal services, while 80% preferred a combination of traditional services. The preference for conventional prenatal care reflects a pragmatic approach among women in rural Zimbabwe. This dual preference allows them to balance the benefits of modern medicine with the cultural sensitivity and accessibility of traditional care. However, reliance on traditional care may pose risks, especially when complications require specialized medical intervention. This is evident in cases where women with health complications were 4.561 times more likely to adopt traditional care, potentially delaying access to life-saving medical services. These findings align with studies in similar contexts, such as Tanzania and Nigeria, where traditional birth attendants (TBAs) are integral due to their affordability, accessibility, and cultural familiarity^5^,^6^. However, this study's findings highlight a context-specific preference for a blended approach, illustrating how traditional and modern healthcare systems can coexist in certain rural settings. This underscores the practical importance of integrated care models in addressing health inequities, while recognizing that healthcare preferences and access may vary across different rural communities in Zimbabwe.

This study highlights the demographic factors influencing traditional prenatal care adoption in rural Zimbabwe. Significant predictors included African Traditional Religion, parity, and low income, reflecting the socioeconomic and cultural realities of rural communities. Women with low income were 9.991 times more likely to use traditional prenatal services, consistent with findings in rural Bangladesh, where financial constraints pushed women toward more affordable care options^15^. These findings align with studies in rural Uganda, where cultural beliefs strongly influence healthcare choices^16^. However, the unique preference for a blended approach underscores the need for integrated maternal care models in Zimbabwe. Education also emerged as a critical factor, with women having only primary education being 5.966 times more likely to adopt traditional care. This aligns with findings from rural India, where lower education levels were associated with reliance on traditional services due to limited awareness of modern care^13^. Contrastingly, predictors like gestation length and religions such as Christianity and Islam were insignificant, suggesting that cultural and socioeconomic factors outweigh clinical or broader religious influences in this context. The findings underscore the complex interplay between demographic, cultural, and socio-economic factors in prenatal care choices. The reliance on traditional care highlights systemic inequities in rural healthcare access, while the preference for integration signals the need for culturally sensitive healthcare solutions.

### Implication for practice

The findings critically impact maternal healthcare policy and programming, particularly in rural Zimbabwe. The high reliance on traditional prenatal services and the preference for a blended approach underscore the need to integrate traditional healthcare practices into formal health systems. Policymakers should consider training and certifying traditional birth attendants (TBAs) to collaborate with conventional healthcare providers. This could enhance the safety and effectiveness of traditional prenatal services while ensuring timely referrals for complications. As suggested in other studies, training community health workers in basic healthcare protocols and emergency recognition should be prioritized to facilitate safe and timely referrals for maternal complications^18^.

The significant influence of demographic factors like low income, education, and parity highlights the need for targeted interventions. Subsidized or free maternal healthcare programs should be expanded to reduce the financial burden on low-income women, who are more likely to rely on traditional care due to cost barriers. Policy makers should allocate resources to subsidize antenatal visits, particularly for low-income and high-parity women, to reduce reliance on unregulated traditional care. Community-based health education campaigns can raise awareness about the benefits of modern prenatal care^19^, particularly for women with lower education levels, while respecting cultural practices.

The strong association between African Traditional Religion (ATR) and traditional care use suggests that health programming should engage local religious leaders and traditional practitioners to promote culturally aligned healthcare messages. Additionally, the preference for combined care models indicates an opportunity to design hybrid prenatal programs incorporating traditional practices within the primary healthcare framework. Finally, addressing systemic rural healthcare challenges—such as improving infrastructure, increasing healthcare worker availability, and reducing distance to clinics—can help bridge the gap between traditional and modern care. These changes can improve maternal health outcomes and reduce risks associated with delayed medical intervention.

## Limitations

This study's cross-sectional design limits its ability to establish causal relationships between the predictors and the adoption of traditional prenatal services. Future research should adopt longitudinal designs to explore how demographic factors influence prenatal care choices over time. Additionally, qualitative studies could provide deeper insights into cultural norms and healthcare accessibility factors influencing maternal care decisions. Additionally, binary logistic regression may not fully capture the complexity of the predictors, as methods like Cox regression could better account for time-to-event aspects of care utilization. The model explained only 48.4% of the variation in traditional care adoption, suggesting that other unmeasured factors—such as cultural norms or healthcare accessibility—may contribute significantly. More so, self-reported questionnaires may come with response biases. To overcome the limitations of self-reporting questionnaires, researchers can incorporate objective measures, such as behavioural observations or physiological data, to enhance the accuracy and reliability of the findings.

## Conclusion

This study highlights the significant influence of demographic factors on adopting traditional prenatal services among rural Zimbabwean women. Age, parity, low income, education, employment, and African Traditional Religion (ATR) were identified as key predictors, with ATR and low income being the strongest influences. The findings reveal a pragmatic preference for integrating traditional care, reflecting the realities of rural healthcare inequities. However, reliance on traditional care in complications raises concerns about delayed medical intervention and associated risks. This study underscores the critical role of demographic factors in shaping prenatal care decisions among rural Zimbabwean women. Policymakers and healthcare providers should prioritize culturally sensitive approaches, subsidized healthcare services, and collaboration with traditional practitioners to address systemic healthcare inequities. Addressing these barriers is essential to improving maternal health outcoes in rural settings.
